# Isotopic Diversity Indices: How Sensitive to Food Web Structure?

**DOI:** 10.1371/journal.pone.0084198

**Published:** 2013-12-31

**Authors:** Anik Brind'Amour, Stanislas F. Dubois

**Affiliations:** 1 IFREMER, Fisheries Ecology and Modelling Department, Ifremer, Nantes, France; 2 IFREMER, DYNECO Benthic Ecology Laboratory, Plouzane, France; Scottish Association for Marine Science, United Kingdom

## Abstract

Recently revisited, the concept of niche ecology has lead to the formalisation of functional and trophic niches using stable isotope ratios. Isotopic diversity indices (IDI) derived from a set of measures assessing the dispersion/distribution of points in the δ-space were recently suggested and increasingly used in the literature. However, three main critics emerge from the use of these IDI: 1) they fail to account for the isotopic sources overlap, 2) some indices are highly sensitive to the number of species and/or the presence of rare species, and 3) the lack of standardization prevents any spatial and temporal comparisons. Using simulations we investigated the ability of six commonly used IDI to discriminate among different trophic food web structures, with a focus on the first two critics. We tested the sensitivity of the IDI to five food web structures along a gradient of sources overlap, varying from two distinct food chains with differentiated sources to two superimposed food chains sharing two sources. For each of the food web structure we varied the number of species (from 10 to 100 species) and the type of species feeding behaviour (i.e. random or selective feeding). Values of IDI were generally larger in food webs with distinct basal sources and tended to decrease as the superimposition of the food chains increased. This was more pronounced when species displayed food preferences in comparison to food webs where species fed randomly on any prey. The number of species composing the food web also had strong effects on the metrics, including those that were supposedly less sensitive to small sample size. In all cases, computing IDI on food webs with low numbers of species always increases the uncertainty of the metrics. A threshold of ∼20 species was detected above which several metrics can be safely used.

## Introduction

The concept of ecological niche has been revisited these recent years with the coming of isotopic ecology and the derived studies on the assessment of the isotopic niche of organisms [1], [2]. Isotopic ecology happily marries Elton's niche concept [3], referring to species trophic interactions and its position in a food web, and Hutchinson's niche concept defined as a *n*-dimensional hypervolume composed of several scenopoetic axes [4]. Adapted to the context of isotopic ecology, Hutchinson's scenopoetic axes are defined in a trophic perspective by isotopes ratios. Stable isotopes (particularly ^13^C and ^15^N) are commonly used to study consumers' trophic pathways providing a time-integrated measure of trophic position, sources of energy, while integrating feeding behaviour and foraging preferences in the mean time [5]. Species isotopic signatures are then represented in a δ^13^C- δ^15^N biplot where species trophic interactions can be qualitatively or quantitatively assessed using a large variety of analytical models or indices [6]. In this kind of graphical representation the species relative positions within the biplot yield information on the resources use, the width of isotopic niche, the species isotopic redundancy, as well as the trophic and/or isotopic diversity of a community.

Following the example of what was done in ecomorphology [7] and to some extent in functional ecology [8]–[11], Layman et al. [12] developed a suite of generic indices describing food webs structure with a focus on the assessment of the trophic niche width and trophic diversity of food webs. Practically, these isotopic diversity indices (IDI) are derived from a set of measures assessing the dispersion/distribution of species in a δ-space. Although thoroughly criticised (see Hoeinghaus et al. [13]), these IDI remain increasingly used in the literature [14]–[18]. The main critics concerned essentially three issues: i) they fail to account for the isotopic overlap of the sources, ii) some indices are highly sensitive to the number of species and/or the presence of rare species, and iii) the lack of standardization prevents any spatial and temporal comparisons. Very few papers have actually studied the sensitivity of the indices to these issues [19], [20].

The sensitivity of the isotopic diversity indices was conceptually assessed by Layman et al. [12] when they first introduced their metrics. The authors qualitatively investigated the behaviour of the metrics when they added three species displaying different isotopic signatures in a community. Very shortly after Layman's paper, Hoeinghaus et al. [13] retorted with a comment in which they criticized the metrics using simple food web structures. They focussed their critics on two issues: failure to account for the sources in the metric's computation, and the necessity to standardise the axes that would allow for spatio-temporal comparison of the metrics. Quantitative studies of the sensitivity of Layman's metrics and particularly the metric assessing the trophic niche width (TA) were done later on with the investigations of Jackson et al. [19] and Syväranta et al. [20]. The two studies used simulations to test the sensitivity of the metrics to the number of species (i.e. low sample size). Jackson et al. [19] also proposed new metrics (Standard Ellipse Areas, SEA and its corrected version, SEAc) apparently unbiased in regards to small sample sizes. They simulated populations with respectively a random mean and a random covariance matrix [19] and a sample mean and associated covariance structure in Syväranta et al. [20]. Therefore, the organisms [19], [20] in both studies were randomly distributed in the δ^13^C- δ^15^N biplots, suggesting a potential random feeding behaviour among them. Only, Jackson et al. [19], in one of their simulations (p.499), drew δ^13^C and δ^15^N values from uniform distributions.

Visual analyses of δ^13^C - δ^15^N biplots suggest that species are not randomly feeding across the food web, they rather use different feeding strategies optimizing their fitness [21]. These foraging strategies could be inferred from the distances and the degree of patchiness between the species' isotopic compositions. For instance, some species could be closely distributed, suggesting direct competition for the resources whereas others could be more distantly distributed suggesting a complementary use of resources or different feeding behavior to limit or avoid inter-species trophic competition. Patchy distribution where species gather together in clusters may thus be the result of different feeding mechanisms. Species are either consuming the same resources (direct trophic competition) or the same functional group of prey (indirect competition), or species are consuming different prey themselves feeding on the same sources (indirect competition). To our knowledge, no studies actually computed and tested the IDI under the hypothesis of feeding preferences, i.e. in a patchy food web. Therefore, our main objective was to investigate using simulations the sensitivity of six commonly used IDI under different trophic food web structures. This was done in regards to the first two critics discussed above: isotopic overlap of the sources and variations in the number of species. We also tested the influence of two types of feeding behaviours: random or selective feeding behaviours. It is worth mentioning that although this study focuses on species, most papers now use TA and SEAc in particular to estimate population niche widths. We advocate that our results and conclusions driven at the scale of the species in a community framework will also prevail at the individual scale in a population framework.

## Materials and Methods

### Simulated food web structures

**Figure 1:**
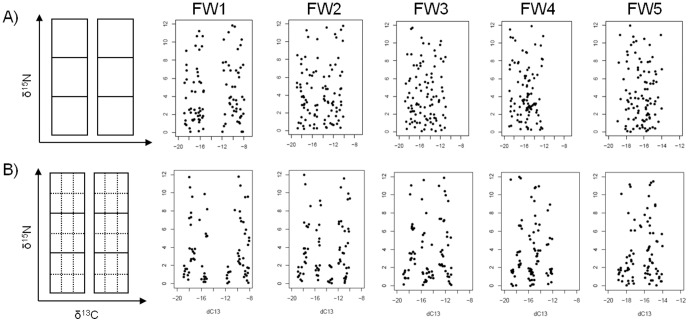
Schematic construction of the simulated food webs under (A) the random and (B) the selective feeding scenario. Codes and description of the different food webs are given in Table 1.

Investigation of the IDI sensitivity to the trophic food web structures was done using simulations on the basis of a large body of literature in isotopic ecology. We first simulated a typical food web in a δ^13^C-δ^15^N biplot and then varied its structure (Table 1 and Figure 1). In the present study, a typical food web basically consists of two food chains (or pathways) with three trophic levels. Each food chain is composed of a varying number of species with a range in δ^13^C of four units (in δ-notation, ‰). For instance, when the two food chains are side by side the total range of δ^13^C spans over eight units (−19‰ to −11‰). While this may seem like a wide range, it is not uncommon to find even greater range between source signatures in nature [22], [23]. Each food chain has three trophic levels ranging in δ^15^N from 0‰ to 12‰. Each trophic level has thus four δ^15^N units. To mimic the pyramid of species in food chains [24], the number of species decreases as we go up in the food chain, so that there are two and four times less species in the second and third trophic level respectively in comparison to the first trophic level. Simulations were then done on a typical food web by varying i) the chains overlap (*i.e.* food chain redundancy), ii) the type of species feeding behaviour (random *vs* selective feeding), and iii) the number of species.

**Table 1:** Ecological properties of the simulated food webs (FW).

Sources overlap	Ecological properties		
**FW1**: Complete distinct basal sources: 3 units of δ^13^C distance between the two food chains		Two distinct trophic food webs supplied by two different basal sources. This is for instance the case in oligotrophic lakes where pelagic and littoral food webs may be completely decoupled [40] or in deep sea ecosystem, where a significant proportion of species in hydrothermal vents assemblages assimilates chemosynthetic material [41].	
**FW2**: Distinct basal sources: 1 unit of δ^13^C distance between the two food chains		Two food chains functioning in parallel in which some species from the different chains may feed on the same basal sources. For example, this situation is reported by Syväranta et al. [18] after a fish removal in a lake. This is also reported in rocky shore community dominated by both suspension-feeders relying on phytoplankton, and grazers relying on macroalgae and epilithon, showing distinct trophic pathways [42] or in estuarine ecosystems when benthic and pelagic communities are decoupled [43].	
**FW3**: Joint basal sources: food chains side by side with 0% overlap		Two joint food chains where some species from the different chains are supplied by the same basal sources. This is notably observed in a Mediterranean deltaic area by Darnaude et al. [44] or in trophic coupling between adjacent benthic habitats [45], [46].	
**FW4**: Partly shared basal sources: 14% overlap between the two food chains		Superimposition of the two trophic food webs with some isotopic redundancy. Several species are either feeding alternatively on the two basal sources or consuming preys themselves preying on the two sources alternatively. This is the case in populations composed of specialist individuals, where a few individuals happened to forage on the same preys [34], [47]. Alternatively, when two primary producers are not discriminated enough to trace distinct trophic pathways in community, with species feeding on a mixture of those sources (*e.g.* suspended particular organic matter and sedimentary organic matter [48]).	
**FW5**: Shared basal sources: 60% overlap between the two food chains		Superimposition of the two trophic food webs where isotopic redundancy reaches its maximum. This case is observed when populations or communities are supplied by organic matter sources not discriminated in δ^13^C such as terrestrial communities grazing on C3 plants only [49] or marine benthic community relying on microphytobenthos and brown macroalgae [50].	

We varied the food chains overlap by positioning the species on a gradient of isotopic redundancy varying from very low redundancy corresponding to two distinct food chains supplied by isotopically differentiated δ^13^C sources, to very high redundancy corresponding to two superimposed food chains sharing the two sources or with two sources with similar isotopic composition. The isotopic gradient is composed of five different states that mimic those observed in nature (Table 1). The types of species feeding behaviour were inspired from the ones commonly found in ecological communities: random and selective feeding. In a recent paper investigating species theoretical species distribution under resources competition, Pigolotti et al. [25] suggested that species experiencing competition would display either a uniform distribution over their resource spectrum or a lump distribution consisting of patches of species sharing similar resources. Therefore we simulated these two feeding behaviours as follow:
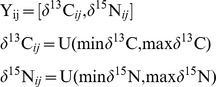
where *i* = 1, 2, … n trophic levels and *j* = 1, 2 food chains.

In the case of random feeding behaviour, each species isotopic signature (Y*_ij_*) was drawn from a uniform distribution with a fixed δ^13^C minimum and δ^13^C maximum for the first food chain and variable δ^13^C minimum and δ^13^C maximum for the second food chain. For the second food chain, the min and max varied with the overlap of the chains (Table 1, Figure 1A). The uniform distributions were computed at each trophic level, that is three times for each food chain with fixed δ^15^N minimum and δ^15^N maximum. We then combined the three levels of each food chain to build up the food web.

Species with selective feeding behaviour were also simulated using values drawn from a uniform distribution with δ^13^C minimum and δ^13^C maximum but instead of creating three uniform distributions per trophic chain we divided each trophic level in six cells and computed uniform distributions in each of the cell (Figure 1B). This created a patchy multimodal distribution. We then combined the three levels of each food chain to build up the food web. Isotopic signatures of each species (Y*_ij_*) were combined as for the random feeding behaviour.

The total number of species (*n*) varies from 10 to 100 every 10 species in the random feeding simulations and from 5 to 80 every ∼8 species in the selective feeding simulations. The number of species is not always a multiple of 10 as the number of species decreases as we go up the food chain. A total of 100 simulations were conducted (*i.e.* 5 sources overlap x 2 feeding behaviours x 10 species richness levels). These simulations were repeated 100 times in order to test the effect of the three factors.

### Simulation assumptions

The simulated food webs have a set of underlying hypotheses. First, all the species within a food chain and within a trophic level display the same feeding behaviour, *i.e.* they are either randomly feeding in the uniform distribution or being selective in the patchy distribution. Second, the trophic enrichment factors were fixed to zero and 4 ‰ for δ^13^C and δ^15^N, respectively. This assumption reflects the theoretical and empirical knowledge [26] that we have on enrichments factors, that nitrogen stable isotope ratios in consumers are typically enriched in the heavier (^15^N) isotope by 2 to 4‰ per trophic level [27], making δ^15^N values useful in defining trophic positions of consumers whereas carbon isotope ratios fractionate to a lesser extent (0 to 1‰) and are typically used to define diet compositions or sources of energy. We are aware that this is a strong hypothesis as recent studies underlined large variability of enrichment factors both within and among populations. Nevertheless, this simplistic assumption should not affect our conclusions as it is not the response *per se* but the relative responses that we were interested in [28].

### Isotopic diversity indices (IDI)

We computed the IDI developed by Layman et al. [12] and the one developed by Jackson et al. [19] estimating the (corrected) niche space of a community (SEAc). The nitrogen range (NR) and carbon range (CR) respectively measure the difference between the δ^15^N and δ^13^C maximum and minimum values. These two metrics were calculated but excluded from the statistical analyses as they were fixed parameters in our simulations. NR which reflects the number of trophic levels was fixed at 12 in all our simulations (i.e. three trophic levels of 4‰). Similarly, CR which reveals the range or differences in basal resources varied between 5 to 11‰ following the simulations (see the *Simulated food web structures* section for details). We computed three IDI assessing the niche space of a community and three others measuring the trophic redundancy and evenness within a food web. The total area (TA) is estimated by the convex hull enclosing all the species. That metric has been largely criticized in the literature [13], [19], [20], notably in regards to its sensitivity to species number. Alternatively, Jackson et al. [19] suggested to assess the niche width using the standard ellipse area (SEA) and the corresponding metric corrected for small sample size (SEAc = SEA * (*n*−1)/(*n*−2), *n* equal to number of species). The centroid distance (CD) is measured as the mean Euclidean distance to the centroid estimated from the average Euclidean distance from each species to the centroid. The (mean) nearest neighbour distance (NND) is estimated as the average of the smallest Euclidean distance between all the species taken two by two. The standard deviation of the nearest neighbour distance (SDNND), supposedly less sensitive than NND to the number of species [12], is computed as the standard deviation of the smallest Euclidean distance between all the species taken two by two. In addition to the latter aforementioned IDI, we computed a composite metric (coefficient of variation of the nearest neighbour distance, CVNND) equalling the standard deviation to mean ratio of the smallest Euclidean distance between all the species taken two by two (i.e. SDNND/NND). This dimensionless measure of dispersion is commonly preferred over the standard deviation to compare the dispersion in different sets of data and particularly in data where the means are considerably different from each other [17], [29]. This metric provides information on the isotopic evenness of the community and as a measure of dispersion, is expected to behave similarly to SDNND and being less influenced than NND by extreme values. Furthermore, CVNND can be interpreted in the same manner as SDNND: low CVNND values suggesting an even distribution of species in the food web.

### Statistical analyses

**Figure 2:**
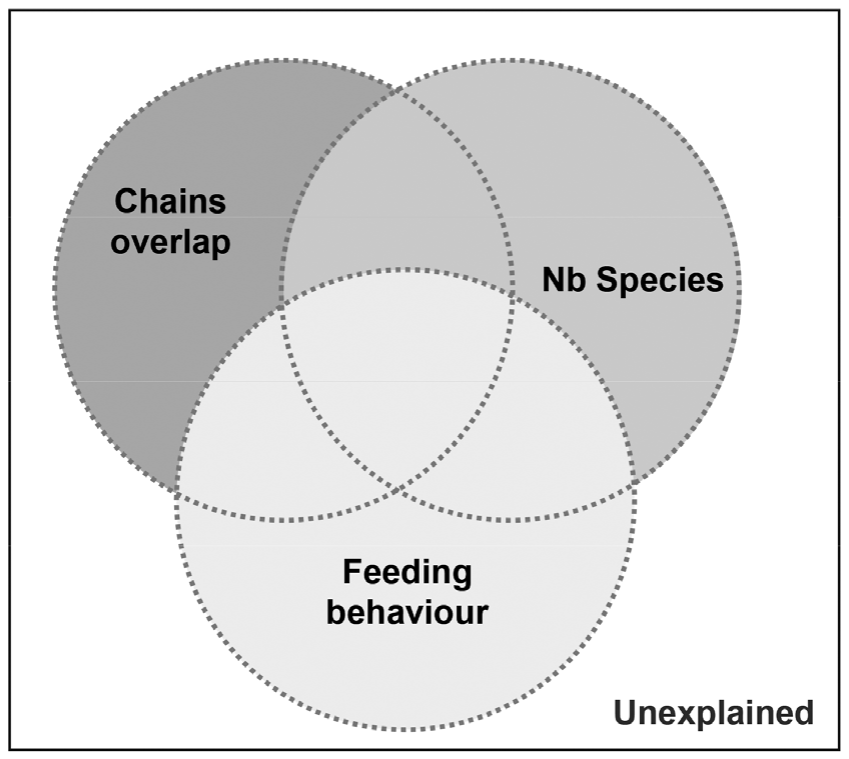
Venn diagram illustrating the pure and the interacting contributions of the three factors tested in this study and the unexplained variance.

Each IDI was calculated for the 10000 simulated food webs corresponding to each of the five chains overlap, two feeding behaviours and the ten species richness levels. We assessed the effect of the three factors on the IDI by variation partitioning [30], [31]. This method consists in partitioning the variance in different fractions assessing unique and combined contributions (*i.e.* interactions) of the factors. Assessment of the different fractions was done by developing seven linear models (LM) for each IDI. Each model is estimating a fraction. For instance, variation partitioning on the SEAc was done as follow. We first estimated the R^2^ from the global model including the three factors. That model gave us the total variance explained by all the factors. Concurrently, 1–R^2^ gave us the unexplained variance. The unique contributions for each factor were estimated by modelling the effect of one factor (*e.g.* chains overlap) while keeping constant the other factors (*i.e.* number of species and feeding behaviour). We ran subsequent models inversing the set of predictors until all the unique and combined fractions have been assessed (Figure 2).

The identification of the minimum number of species required to avoid the bias associated with small sample size was done by estimating inflexion points. The minimum number of species was identified on each IDI-species richness relationships: when the curve was visually reaching a plateau (*i.e.* asymptotes to a constant), the minimum number of species was estimated using the first inflexion point of the curve. The inflexion point was assessed in three steps: i) fitting a cubic smoothing spline on the data, ii) computing the second derivative of the smoothed data, and iii) identifying the first value equal to zero. These steps were done for each IDI of the random and selective feeding behaviours (whenever the IDI-species curve reached an asymptote). Assessment of the inflexion point was done on the overall dataset of each ID (i.e. combining the five responses of the chain overlap), as they displayed similar responses with the number of species (see details in the results). All the figures and statistical analyses were conducted in R language [32].

## Results and Discussion

**Figure 3:**
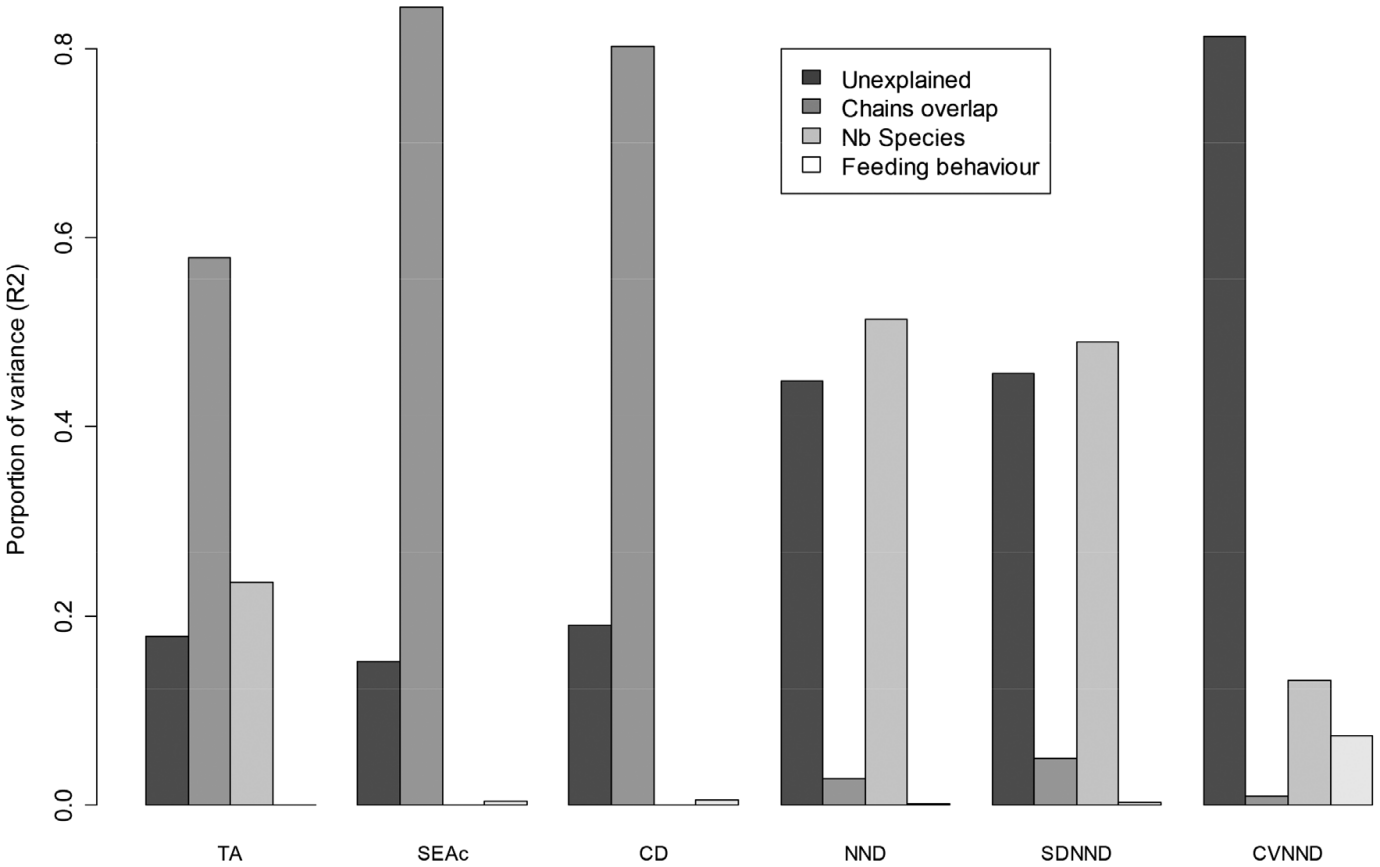
Proportion of variance explained by the pure contributions of the three studied factors and the unexplained variance. Codes for the indices are described in the text.

Simulations on the community metrics suggested that all the IDI are sensitive to either one or several factors tested in here, *i.e.* chains overlap, number of species and to a lesser extent the feeding behaviour of the species. The IDI are not equally influenced by these factors (Figure 3) and we aimed at identifying the conditions and limits under which they can be safely used in isotopic ecology.

### Sensitivity to chains overlap

**Figure 4:**
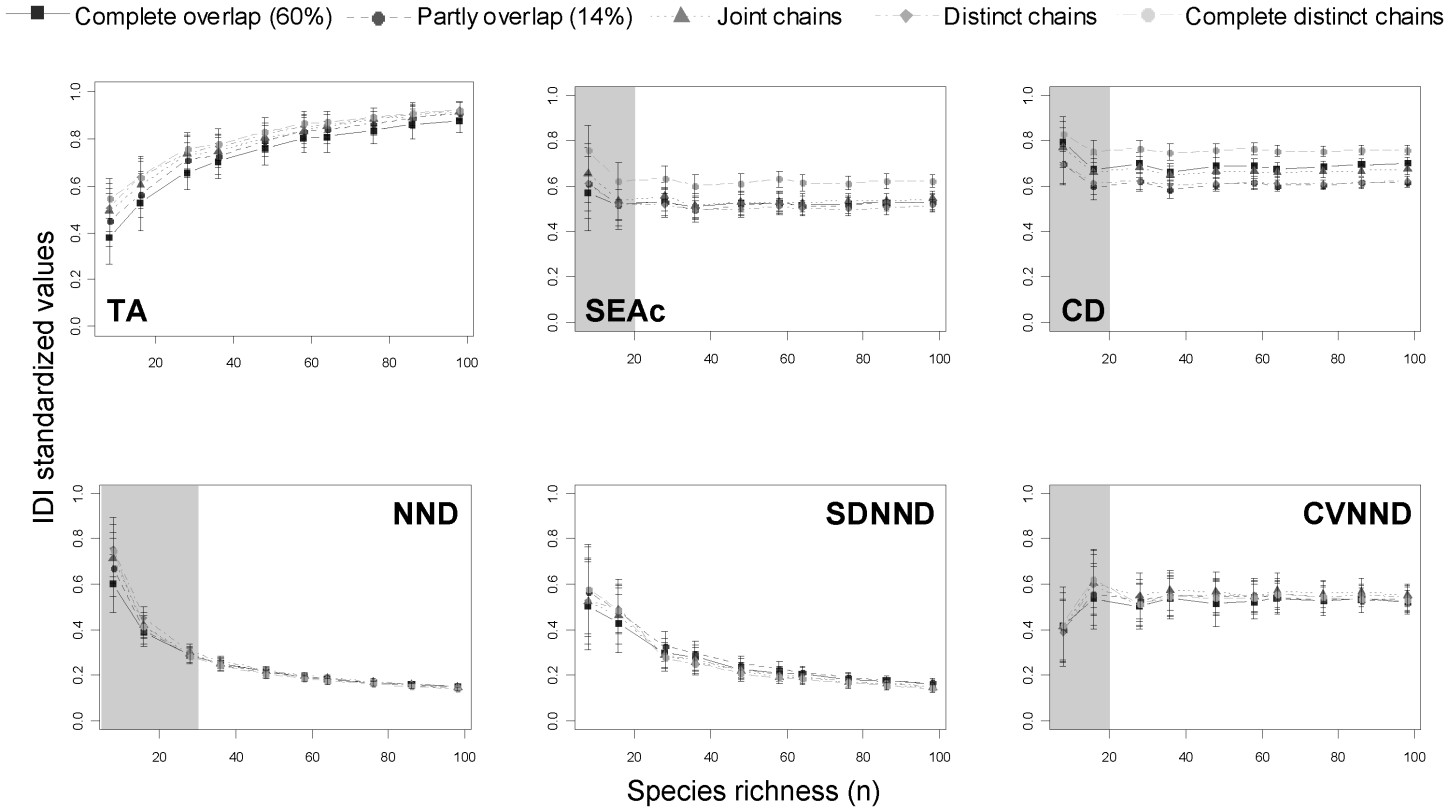
Isotopic diversity indices (IDI) calculated using simulated data under the random feeding scenario. The bars indicate the standard deviation estimated from 100 repetitions. The different types of lines define the degree of chains overlap. Reported values of IDI are standardized by dividing each IDI by the maximum of that IDI. Shaded areas underline the minimum number of species required to avoid small sample size bias.

**Figure 5:**
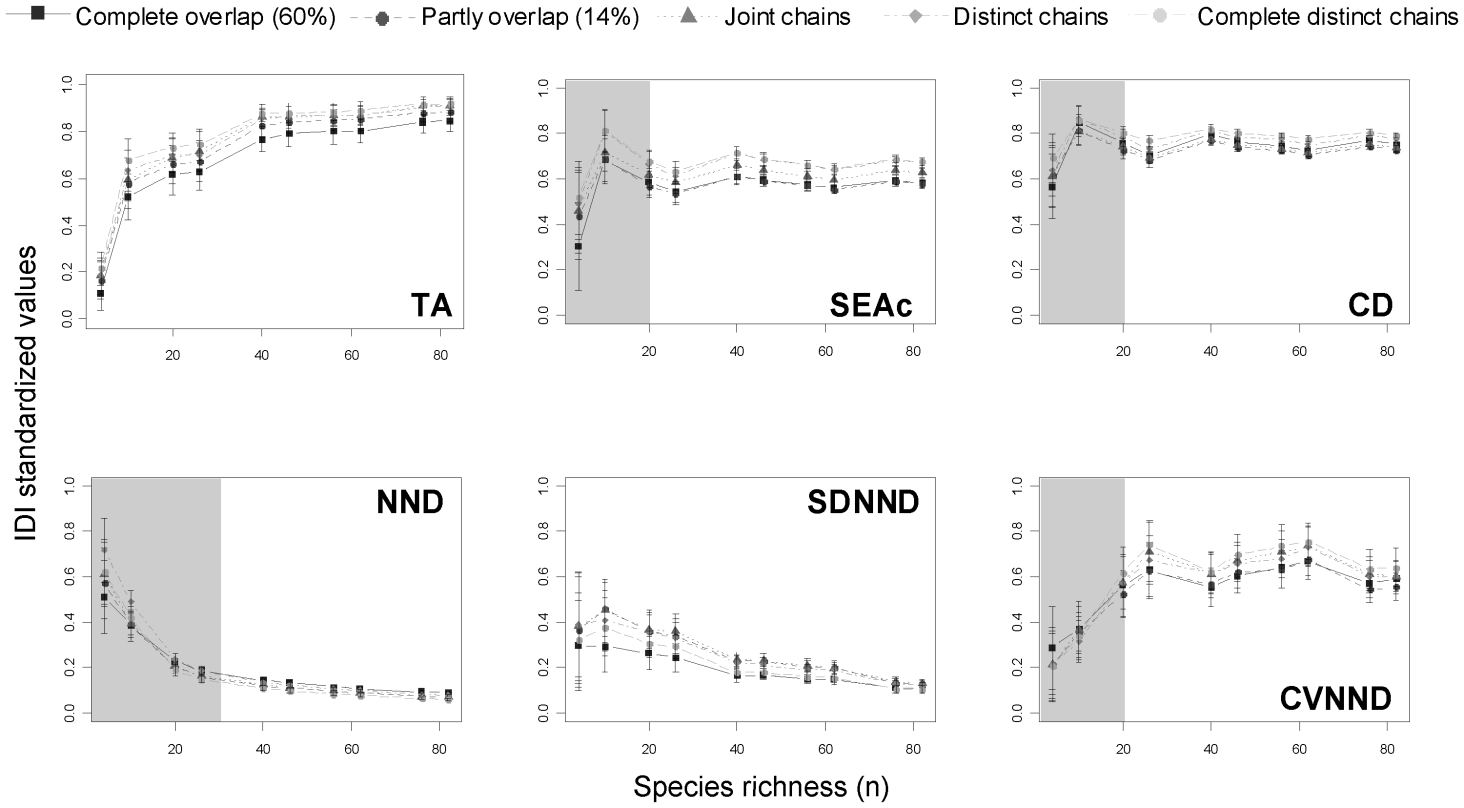
Isotopic diversity indices (IDI) calculated using simulated data under the selective feeding scenario. The bars indicate the standard deviation estimated from 100 repetitions. The different types of lines define the degree of chains overlap. Reported values of IDI are standardized by dividing each IDI by the maximum of that IDI. Shaded areas underline the minimum number of species required to avoid small sample size bias.

The three most sensitive IDI to the degree of chains overlap were the TA, SEAc and CD (see “chains overlap” bars in Figure 3). These three metrics are conceptually very different as the first two measure a surface indicating the trophic niche width or space while the third one (CD) is a distance revealing species trophic redundancy. TA and SEAc are two measures highly sensitive to variations in δ^13^C and δ^15^N ranges. The range of δ^15^N was held constant in this study (*i.e.* no changes in food chain length were tested), thus the responses of these metrics are exclusively interpretable on the δ^13^C variations, which corresponds to changes in primary producers isotopic compositions supporting the food chains. TA values decreased with the degree of chains overlap in both the random (Figure 4) and selective feeding scenarios (Figure 5), although in the latter scenario the complete overlap situation tends to differ from the others. For instance, in the random feeding scenario, completely distinct chains gave TA values 2.5 times higher than completely overlapping chains (60% of overlap). This amplification is coherent with what was expected: food webs based on isotopically different sources or displaying different pathways should yield larger trophic niche space (i.e. TA values), as the species ultimately may feed on a larger spectrum of preys/resources [18].

The SEAc values also decreased with the overlap of the chains but contrary to TA, where the values varied almost linearly with the degree of chains overlap, SEAc showed a threshold below which no differences were observed. That threshold varied according to the feeding scenario. In the random feeding scenario, the threshold was located between the complete distinct chains and the four others (Figure 4), suggesting a potential underestimation of the niche space in communities for example, feeding in different habitats [17], [23] or belonging to different size classes, themselves feeding on isotopically well differentiated sources [33]. In the case of selective feeding scenario, likely closer to reality [25], SEAc displayed three groups of responses: higher values for complete distinct or distinct sources, intermediate values when the two food chains are joint, and smaller values in the case where the two sources overlap either partially or completely (Figure 5). The inability of the SEAc to differentiate among the two extreme categories (complete distinct and distinct overlap or partly overlapped andcompletely overlapped) remains unexplained but models reveal here that in certain configurations (*e.g.* differences in δ^13^C≈3‰), SEAc may slightly underestimate the niche space of the community.

The CD varied greatly with the chains overlap in the random feeding scenario but very little in the selective one. Just as in the case for the TA and SEAc, it was expected that the highest values of CD would be in the case where the two food chains are distinct (larger range of δ^13^C). However, intermediate CD values in complete overlap and joint chains and smaller values in the case of partly overlap and distinct chains is unexpected and unexplained (Figure 4).

The last three IDI computed in this study are by-products of the smallest distance between two neighbours and were less sensitive to the overlap of the chains. The NND did not show any differences among the chains overlap in both scenarios whereas the SDNND and CVNND were more sensitive in the selective feeding scenario (as shown by the distance between the curves and the large error bars in Figure 4). In that case, increasing the degree of overlap between the two trophic chains does not seem to increase the risk of revealing higher trophic competition between species exhibiting close isotopic signatures. For example, such situation is encountered in populations composed of specialists: several individuals occupy similar specialized trophic niches (high redundancy) but the population appears to have very limited food competition [34]. Our results revealed that changes in food sources isotopic composition towards a decrease in δ^13^C ranges may affect the CVNND.

### Sensitivity to species number

Small sample sizes give disproportionate weights to species displaying extreme values of δ^13^C and/or δ^15^N and all the IDI measured in this study were to a certain extent sensitive to this factor. SEAc, CD and to a lesser extent, CVNND, were less impacted by small sample sizes in random feeding scenario (Figure 4). SEAc is estimated on a sample of species containing nearly 40% of the species rather than on the entire community (see Jackson et al. [19] for details), it was thus expected to be less influenced by small numbers of species. The CD explicitly recompile a centre of gravity (i.e. centroid), thereby reducing the influence of extreme values. SEAc and CD are both mathematically close to one another as they are both based on the dispersion of measurements around a centroid and they reflect the “core” of the niche rather than the “complete” niche space [15]. Not surprisingly the two metrics behave in the same way under the various scenarios in our simulations. As for CVNND, it remained almost unchanged by the number of species in the random feeding scenario but tended to overestimate the evenness in small sample size when species displayed feeding preferences (Figure 5). The constancy of that metric notwithstanding the species number suggests a certain stability that was not found in the SDNND. Layman et al. [12] acknowledged that SDNND would be less influenced by small sample size than NND. In our simulations, SDNND was indeed quantitatively less impacted than the NND by the number of species, but it was still underestimating the evenness in the community of small sample size (Figure 4 and 5). Similar results were reported by Jackson et al. [19] in their supplementary material where they studied the responses of the same two metrics to species number. Although the authors simulated a range of species varying from 1 to 1000 species, they showed that within the range of our simulations (1 to 100 species) the metric values are exponentially decreasing with increased sample size.

Larger uncertainty was found for all the metrics with small sample size (Figures 4 and 5), confirming once again that they are all sensitive to extreme values. For certain metrics (e.g. SDNND) the uncertainty in a given scenario for small sample size (e.g. complete chains overlap in selective feeding scenario) could exceed the range of values reached by that same metric at different (but larger) sample size. Based on these results and the stability of the CVNND in comparison to SDNND, we suggest the use of CVNND to assess the trophic evenness of a community. Interestingly, Quevedo et al. [17] computed an adapted version of that metric (*i.e.* distance between an individual to all neighbours rather than the distance between the two closest neighbours). Even though these authors discussed their adaptation, they did not explain why they preferred the coefficient of variation rather than the standard deviation. We presume that they used the CVNND for the same reasons as evidenced here.

For IDI reaching an asymptote at a constant value, we estimated the minimum number of species before reaching that asymptote (*i.e.* inflexion point). Four metrics corresponded to that criterion: CD, SEAc, CVNND, and NND. Estimation of the inflexion point suggested that for the first three metrics, a minimum of 20 species (or individuals) is required to avoid any bias associated with small sample size (see shaded areas in Figures 4 and 5), whereas we estimated a minimum of 30 species for the NND. These results agree very well with conclusions from Syväranta et al. [20].

### Sensitivity to feeding behaviour

Communities are generally composed of species exhibiting different foraging strategies allowing them to access preferential food items thereby maximising both nutritional and reproductive efficiency [21], [23]. These feeding preferences inspired our selective feeding scenario and were translated in the δ-space by patches of species displaying similar isotopic signatures. We acknowledge that species being closely located in the δ-space may result from either similar prey selection or from a mix of prey individually displaying different isotopic signature but altogether displaying similar isotopic signatures. As we simulated five different scenarios of overlapping chains (*i.e.* different pathways), we believe that our results may be best interpreted in the context of feeding preferences. To our knowledge, the studies that quantitatively tested the robustness of the IDI simulated random distribution of organisms [19], [20]. In the random scenario of this study, results strongly agree with their findings; TA behave badly with low sample size and SEAc may correctly estimate the isotope niche width, given a minimum number of species but with some uncertainty [20]. The originality of this work primarily resides in testing the influence of a situation that mimics more closely the reality, that is a patchy distribution of species in a δ-space. Thus, visual comparison of the curves in Figure 4 and Figure 5 highlights very large uncertainties in the selective feeding scenario for all the IDI, except the NND which displayed constantly low variability notwithstanding the feeding behaviour. Quantitative contribution of the feeding behaviour to the IDI responses assessed by the variance partitioning indicates that only the CVNND was slightly affected by the selective feeding behaviour (Figure 3). Interestingly, this ratio allows us to detect some variation where NND and SDNND did not varied according to changes in feeding behaviour.

### Facets of trophic diversity

Structural diversity can be split in two different components: species richness which is a simple count of species and species evenness which quantifies how equal the abundances of the species are [35]. Similarly, Mason et al. [10] and Mouillot et al. [36] suggested to split and measure the functional diversity into three components: functional richness which quantifies the amount of niche space filled by a trait in the traits community space, functional evenness which identifies how species traits are distributed within that space (*i.e.* regularity), and functional divergence which specifies the position and degree of clustering of the traits within that space (clustered near the edge or clustered in centre). In a recent study, Villéger et al. [37] suggested to decompose the trophic diversity into the same functional diversity components: trophic richness, trophic evenness, and trophic divergence. They adapted and computed functional diversity metrics [11] into a “trophic space” using the trophic level as a species functional trait. Using the metrics of the present study we suggest, as a complement to Villéger et al. [37], the following decomposition of the trophic diversity in three components: i) the *Trophic richness* describes the niche space species occupy in the δ-space. This component is measured by CR, NR, but more specifically by TA, SEAc (and SEA), and CD; ii) the *Trophic redundancy* represents the overall species packing and is assessed by the average of the smallest distance between two points (NND) and also to some extent by CD. Small values indicate dense patches of species displaying similar isotopic signature, thus likely feeding on the same isotopic prey; and iii) the *Trophic evenness* reports how regularly distributed are the species in the δ-space. The SDNND and CVNND are both assessing that component. Low values indicate an even distribution of species within the food web. As opposed to the adapted metrics of Villéger et al. [11], the metrics used in our study are not weighted for species abundance or biomass, yet conferring equal importance to all the species in the food web and precluding the computation of Villéger's *Trophic divergence*.

## Conclusion

Our work and particularly the simulations under the random feeding scenario confirms the findings of other recent studies assessing the sensitivity of Layman's diversity indices that two of them (TA and NND) are quite sensitive to the number of species. This conclusion was also reached using our second scenario of selective feeding behaviour. However in that scenario, which in our point of view is more realistic than the random scenario, the large uncertainties displayed by all the IDI - even in high sample size - raise the alarm when one is comparing food web structures.

Based on the IDI tested in this paper, if one is interested in estimating the niche space of a community or population we suggest to use SEAc or CD. Nevertheless, the use of SEAc should be done cautiously, as we demonstrated that a minimum number of 20 species (or individuals) should be sampled. Below that threshold the IDI values may be over or underestimated. Other metrics not tested in here but that have been proved unbiased in respect to species number include those developed by Bolnick et al. [38] as well as circular statistics [39]. The recent paper of Syväranta et al. [20] showed that the computation of some IDI could be used when dealing with populations instead of communities. Concurrently, results from our study are easily applicable and interpretable at the scale of the species. When the IDI are indeed computed at the species scale, they measure the individual feeding diversification of a species and estimate the trophic niche width or the redundancy and evenness within species. We advocate that more critical examinations of isotopic diversity metrics are needed in order to really understand how they perform in real life data and bring new insights into their ecological meaning.
